# Temporal patterns and geographic heterogeneity of Zika virus (ZIKV) outbreaks in French Polynesia and Central America

**DOI:** 10.7717/peerj.3015

**Published:** 2017-03-21

**Authors:** Ying-Hen Hsieh

**Affiliations:** Department of Public Health and Center for Infectious Disease Education and Research, China Medical University, Taichung, Taiwan

**Keywords:** ZIKV, Oceania, Central Aermica, Geography, Temporal, Heterogeneity, Phenomenological model, Basic reproduction number, Turning point

## Abstract

**Background:**

Zika virus (ZIKV) transmission has been reported in 67 countries/territories in the Oceania region and the Americas since 2015, prompting the World Health Organization (WHO) to declare ZIKV as a Public Health Emergency of International Concern in February 2016, due to its strong association with medical complications such as microcephaly and Guillain–Barré Syndrome (GBS). However, a substantial gap in knowledge still exists regarding differing temporal pattern and potential of transmission of ZIKV in different regions of the world.

**Methods:**

We use a phenomenological model to ascertain the temporal patterns and transmission potential of ZIKV in various countries/territories, by fitting the model to Zika case data from Yap Island and French Polynesia in the Oceania region and 11 countries/territories with confirmed case data, namely, Colombia, Ecuador, French Guiana, Guadeloupe, Guatemala, Mexico, Nicaragua, Panama, Puerto Rico, Saint Martin, and Suriname, to pinpoint the waves of infections in each country/territory and to estimate the respective basic reproduction number *R*_0_.

**Results:**

Six of these time series datasets resulted in statistically significant model fit of at least one wave of reported cases, namely that of French Polynesia, Colombia, Puerto Rico, Guatemala, Suriname and Saint Martin. However, only Colombia and Guatemala exhibited two waves of cases while the others had only one wave. Temporal patterns of the second wave in Colombia and the single wave in Suriname are very similar, with the respective turning points separated by merely a week. Moreover, the mean estimates of *R*_0_ for Colombia, Guatemala and Suriname, all land-based populations, range between 1.05 and 1.75, while the corresponding mean estimates for *R*_0_ of island populations in French Polynesia, Puerto Rico and Saint Martin are significantly lower with a range of 5.70–6.89. We also fit the Richards model to Zika case data from six main archipelagos in French Polynesia, suggesting the outbreak in all six island populations occurred during the same time, albeit with different peak time, with mean *R*_0_ range of 3.09–5.05.

**Discussion:**

Using the same modeling methodology, in this study we found a significant difference between transmissibility (as quantified by *R*_0_) in island populations as opposed to land-based countries/territories, possibly suggesting an important role of geographic heterogeneity in the spread of vector-borne diseases and its future course, which requires further monitoring. Our result has potential implications for planning respective intervention and control policies targeted for island and land-based populations.

## Introduction

Zika virus (ZIKV), a flavivirus, has been known since its first isolation from a primate in 1947 in the Zika forest of Uganda, and a year later in 1948 from *Aedes africanus* mosquitos in the same location (Dick, Kitchen & Haddow, 1952). Serological evidence of ZIKV infection in humans has been reported since 1951, but it had been confined to the equatorial regions of Africa and Asia until recently (Pierson & Diamond, 2013). Moreover, *Aedes aegypti* mosquitos have been found to be the primary transmitter of Zika virus in human populations in the more recent outbreaks in the Americas (Monaghan et al., 2016).

Available scientific evidence strongly suggests that Zika virus causes Guillain–Barré Syndrome (GBS) (Cao-Lormeau et al., 2016; De Paula Freitas et al., 2016). Moreover, Zika infection in pregnant women is reported to associate with, among other medical complications, microcephaly in their infants (Brasil et al., 2016; Cauchemez et al., 2016), fetal deaths, stillbirths, and central nervous system lesions (Sarno et al., 2016). As of August 25, 2016, a total of 466,815 suspected and 111,333 confirmed autochthonous transmission cases have been reported in 47 countries/territories in the Americas from Mexico to Argentina, with 10 deaths among the reported cases (Pan American Health Organization, 2016a). Moreover, Zika virus (ZIKV) transmission has been reported in 67 countries/territories in the Oceania region and the Americas since 2015. Currently without vaccines or medication for treatment, the global Zika outbreaks have prompted the World Health Organization (WHO) to declare ZIKV as a Public Health Emergency of International Concern in February 2016 (World Health Organization, 2016).

Several recent modeling studies have been carried out to investigate the transmission potential and temporal patterns of Zika outbreaks in countries and territories in the Americas and Oceania. In this work, we will make use of a simple phenomenological model, the Richards model, to fit the epidemic data of 13 countries/territories in the Americas and Oceania in an attempt to obtain a more comprehensive understanding of the outbreaks in each country, in terms of their similarities and differences in relation to their respective geographic locations and characteristics.

## Methods

### Data

The data for Yap Island and French Polynesia in the Oceania region used in this study are obtained from published literature (Duffy et al., 2009; Ioos et al., 2014; Centre d’hygiène et de Salubrité publique, 2014). The former is the weekly laboratory confirmed case data from April 1 to July 29, 2007 in Yap Island (Duffy et al., 2009), while the latter data is that of weekly number of suspected Zika cases in six main archipelagos (Tahiti, Iles sous-le-vent, Moorea, Tuamotu-Gambier, Marquises, and Australes) in French Polynesia, from October 30, 2013 to March 28, 2014 (Ioos et al., 2014; Centre d’hygiène et de Salubrité publique, 2014). For the Zika outbreak data in the Americas, we make use of the reported weekly Zika case data of Pan American countries and territories from ZIKV epicurves provided by the Pan American Health Organization (PAHO) website (Pan American Health Organization, 2016b, accessed on June 7, 2016). For most countries, both confirmed and suspected data are given. Here we choose to use the confirmed case data only. More recently updated data from some PAHO countries/territories are also accessed from the PAHO website (Pan American Health Organization, 2016c). Data used in this study with model fit are provided in Table S1.

### Mathematical model

In order to identify a wave of infections during an infectious disease outbreak, we make use of the analytic solution of the Richards growth model (Richards, 1959) of the form

\begin{eqnarray*}C(t)=K[1+{e}^{-ra(t-{t}_{i}-(\ln \nolimits a)/ra)}]^{-1/a}, \end{eqnarray*}

where *C*(*t*) is the cumulative number of Zika cases at day *t*, and *t* = 0 is the starting week of the wave. *K* is the total case number of the wave, *r* is the per capita growth rate of the cumulative case number, *a* is the exponent of deviation of the cumulative case curve, and *t*_*i*_ is a turning point which signifies the exact moment of an upturn or downturn in the rate of increase for the cumulative case number (Hsieh, Lee & Chang, 2004; Hsieh & Cheng, 2006).

The Richards model is a phenomenological model which describes the growth of the cumulative case number. *K*, *r*, and the turning point *t*_*i*_ are three model parameters of epidemiological importance. These parameters can be estimated by fitting the Richards model to the epicurve of the outbreak, using standard software with nonlinear least-squares (NLS) approximation subroutines, e.g., SAS MATLAB, or R. (SAS code provided in Table S2.). The criterion for a good model fit is that the NLS estimation converges with *p*-value < 0.05 and that the 95% confidence intervals (CI) for all estimated parameters are positive, in order to ensure that all estimated model parameter values are significant. If a wave is achieved, we repeat the fitting procedure starting from the endpoint of this wave to attempt to obtain a subsequent wave, to see if it indeed exists. Readers are referred to Hsieh & Cheng (2006) for more details on fitting of multi-stage Richard model.

### Reproduction number

The basic reproduction number *R*, the average number of secondary infectious cases produced by an infectious case in a totally susceptible population in the absence of interventions is *R*_0_ = exp(*rT*), where *r* is the per capita growth rate from the Richards model and *T* is the serial interval of the disease or the average time interval from onset of one individual to the onset of another individual infected by him/her. It has been shown mathematically (Wallinga & Lipsitch, 2007) that, given a growth rate *r*, the expression *R*_0_ = exp(*rT*) provides an upper bound for the basic reproduction number over estimates that can be obtained from all assumed distributions of the serial interval *T*. For this study, we let the mean of *T* = 16 days with a range of 10–23 days as proposed in Majumder et al. (2016a) and Majumder et al. (2016b).

## Results

We fit the Richards model to time series data of Zika case number from Yap Island (2007), French Polynesia (2013–2014), and 11 countries and territories in Latin America with significant number of weekly confirmed Zika cases from 2015 up to week 18 of 2016, namely, Colombia, Ecuador, French Guiana, Guadeloupe, Guatemala, Mexico, Nicaragua, Panama, Puerto Rico, Saint Martin, and Suriname.

**Figure 1:**
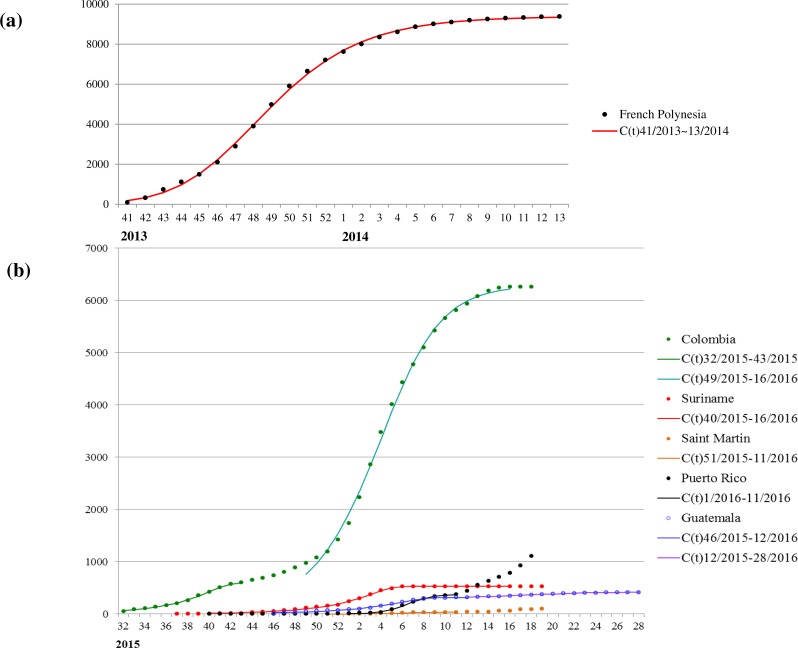
Model fit for weekly cumulative Zika case number in: (A) French Polynesia, week 41, 2013–week 8, 2014; (B) Colombia, Suriname, Guatemala, Saint Martin, and Puerto Rico, week 32, 2015–week 11, 2016.

**Figure 2:**
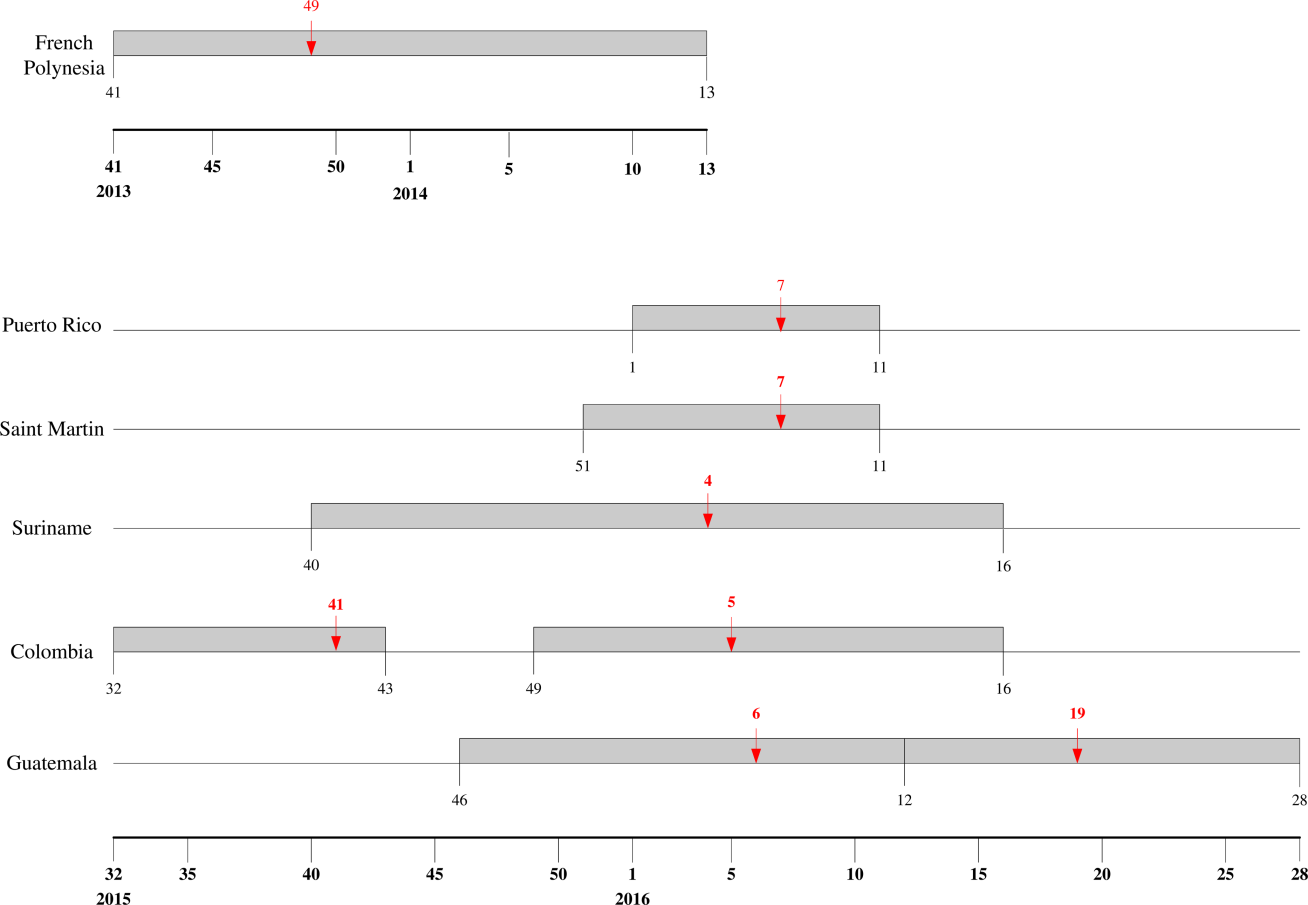
Timeline for ZIKV outbreaks in French Polynesia, Puerto Rico, Saint Martin, Suriname, Guatemala, and Colombia. Red arrow denotes the turning point.

The mean estimates of the model parameters from model fit, as provided in Table 1, show that only six of these 13 time series data, namely, French Polynesia, Colombia, Guatemala, Puerto Rico, Saint Martin and Suriname, can provide good fit with the Richards model with at least one distinct wave of cases. In particular, only Colombia and Guatemala have two waves of cases while all other data fitted only result in one wave. The model fits for these six countries/territories are given in Fig. 1. The 95% CI from model fitting by SAS is provided, except those of the basic reproduction number *R*_0_, which are computed from the expression for *R*_0_ using the 95% CI range of the estimate for *r* and the range of [10, 23] for *T*. We also give the adjusted *R*^2^ (see, e.g., Theil, 1961) as a measure of the goodness of fit. For the purpose of comparing temporal trends and the level of synchronicity of these six countries/territories, the timelines of the waves of outbreaks are given in Fig. 2.

**Table 1:** Summary of parameter mean estimates for weekly Zika case fitting of French Polynesia, Colombia, Suriname, Guatemala, Saint Martin, and Puerto Rico with the Richards model, with 95% confidence interval in parenthesis.

Country	Time period	*r*	*K*	*t*_*i*_	*R*_0_	Adjusted *R*^2^
French Polynesia	41/2013–13/2014	0.78 (0.49,1.08)	9380 (9286,9473)	7.1 (6.8,7.4)	6.00 (0.06,11.95)	0.999
Colombia	32/2015–43/2015	0.25 (0.22,0.27)	619 (571,668)	8.1 (7.6,8.5)	1.75 (1.34,2.16)	0.998
	49/2015–16/2016	0.26 (0.19,0.32)	6288 (6110,6465)	7.1 (6.4,7.8)	1.79 (1.29,2.30)	0.998
Suriname	40/2015–16/2016	0.23 (0.21,0.24)	528 (523,532)	15.2 (14.8,15.6)	1.68 (1.32,2.04)	0.999
Guatemala	46/2015–12/2016	0.20 (0.18,0.22)	316 (306,326)	12.0 (11.3,12.7)	1.59 (1.28,1.90)	0.997
	12/2016–28/2016	0.023 (0.021,0.025)	412 (409,416)	6.5 (4.4,8.7)	1.05 (1.03,1.08)	0.997
Saint Martin	51/2015–11/2016	0.76 (0.42,1.11)	30 (29,32)	7.0 (6.5,7.6)	5.70 (0^*^,11.75)	0.996
Puerto Rico	1/2016–11/2016	0.84 (0.36,1.33)	375 (351,400)	5.4 (5.0,5.9)	6.89 (0^*^,16.24)	0.998

**Notes.**

* max(lower bound, 0).

**Figure 3:**
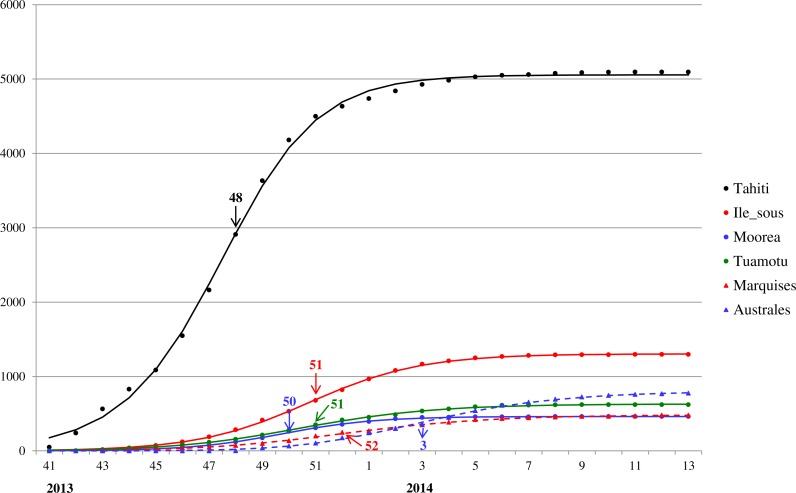
Model fit for weekly cumulative Zika case number in Tahiti, Iles sous-le-vent, Moorea, Tuamotu-Gambier, Marquises, and Australes in French Polynesia, week 41, 2013–week 13, 2014. The turning points are indicated with colored arrows.

For a closer look at Zika outbreak in island populations, we also fit the Richards model to the weekly number of suspected Zika cases in the six main archipelagos in French Polynesia, namely, Tahiti, Iles sous-le-vent, Moorea, Tuamotu-Gambier, Marquises, and Australes, from October 30, 2013 to March 28, 2014 (Centre d’hygiène et de Salubrité publique, 2014). The results of the fittings are given in Table 2 and Fig. 3.

**Table 2:** Summary of parameter mean estimates for weekly Zika case fitting of six archipelagos in French Polynesia with the Richards model, with 95% confidence interval in parenthesis.

Region	Time period	*r*	*K*	*t*_*i*_	*R*_0_	Adjusted *R*^2^
Tahiti	41/2013–13/2014	0.49 (0.39,0.60)	5056 (5009,5103)	6.5 (6.2,6.9)	3.09 (1.49,4.68)	0.999
Iles sous-le-vent	41/2013–13/2014	0.49 (0.43,0.56)	1305 (1295,1314)	9.7 (9.5,9.9)	3.08 (1.60,4.57)	1.000
Moorea	41/2013–13/2014	0.60 (0.48,0.72)	463 (459,467)	8.7 (8.5,9.0)	3.92 (1.49,6.34)	0.999
Tuamotu-Gambier	41/2013–13/2014	0.49 (0.38,0.61)	630 (622,638)	9.3 (9.0,9.7)	3.09 (1.45,4.73)	0.999
Marquises	41/2013–13/2014	0.49 (0.32,0.66)	485 (474,495)	10.9 (10.4,11.3)	3.08 (1.24,4.92)	0.999
Australes	41/2013–13/2014	0.71 (0.37,1.04)	804 (780,827)	13.7 (13.3,14.1)	5.05 (0^*^,10.14)	0.999

**Notes.**

* max(lower bound, 0).

## Discussion

### Temporal patterns

Among these six countries/territories, French Polynesia and Colombia have the largest outbreaks reported. However, their respective characteristics are decidedly different. Comparing the second, larger wave in Colombia with the outbreak in French Polynesia, the latter outbreak had more than twice the case number and much higher transmissibility (as quantified through their respective estimates of basic reproduction number *R*_0_), but with very similar temporal patterns as exhibited by the exact same length of the wave of cases (20 weeks) and by the comparable timing of the turning point (6.4 weeks after the start of the second wave in Colombia vs. 7.1 weeks after for French Polynesia).

It is interesting to note that for the first wave in Colombia, a downward turning point of 8.1 pinpointed the week of October 11–17 in 2015, during which nine samples were laboratory-confirmed as Zika virus infection in Colombia, which happened to be the first cases of Zika virus infection detected in the country (World Health Organization, 2015). Therefore, the turning point reflects the reporting of this cluster of cases. We also note that multiple waves are also frequently observed in dengue outbreak, another vector-borne disease that also spread via *Aedes aegypti* mosquitos and have been found to have some similar epidemiologic characteristics under the same setting, such as basic reproduction number (Funk et al., 2016).

The model fit for the six island populations in French Polynesia reveals synchronous waves between week 41, 2013 to week 13, 2014. The fitting results, shown in Fig. 3 and Table 2, indicate the waves on these islands occurred simultaneously in timing but with very different turning points. The turning points for the waves vary substantially from week 48 of 2013 in Tahiti (6.5 weeks after week 41) to week 3 of 2014, a range of almost 2 months (see Fig. 3). Since the turning point often coincides with the peak time of incidence, this discrepancy indicates that, although the outbreak had occurred during the same time in all six archipelagos, it had peaked and hence declined at different times, perhaps suggesting a disparity in response/intervention. However, from Fig. 3 it appears that Tahiti might be missing data from early stage of the wave before week 41, and hence is likely the earliest wave when compared with the other archipelagos. This observation is also consistent with the early turning point pinpointed for Tahiti compared to those of the archipelagos.

### Geographic Heterogeneity

The wave of cases in Suriname follows a very similar pattern as the waves in Colombia, albeit slightly starting and ending later. Even the turning point of the wave of infection in Suriname came only one week earlier than that of second wave in Colombia, indicating very similar temporal patterns (see Fig. 2). Moreover, the transmissibility is also similar, with very comparable ranges of *R*_0_ for the two countries, which is surprising since these two countries have no common border, with Venezuela, French Guiana and Brazil in between. The two waves of cases we detected for Colombia have similar range of *R*_0_. Although *R*_0_ is slightly smaller for the second wave, it has a substantially larger number of cases, as it has been often observed that having higher transmissibility does not imply a more sizable outbreak (Hsieh, De Arazoza & Lounes, 2013). For Guatemala, however, *R*_0_ for the second wave is significantly lower than that of the first wave.

The two Caribbean island countries/territories, Puerto Rico and Saint Martin, also stand out in drawing close comparison in their respective wave of reported Zika cases. Other than the discrepancy in case number (which again might be due to the difference in population sizes), the respective waves are almost synchronized in time with exactly the same week for a turning point, and with similarly high transmissibility and wide 95% CI ranges.

We have refrained from using the suspected case data for our model fitting, based on the distinctly different nature of confirmed and suspected case data. While the confirmed case data are typically confirmed either clinically or in laboratory and could underreport the true symptomatic cases, the suspected case data often contain cases that cannot be confirmed and hence tend to overstate the true magnitude of outbreaks and perhaps significantly distort the temporal growth in the cumulative data that is used for fitting of the Richards model. Chowell and others (2016) used suspected Zika data in Colombia during January–April 2016 and a generalized Richards model to obtain mean estimates of reproduction number of 2.2 and 10.3, which is higher than our result for the second wave that overlaps the time period of their dataset. The substantial difference might be a consequence of the difference in outcome that might result from using confirmed or suspected cases. In Table 3, we provide a summary of previous results on the estimates of *R*_0_ in literature, although the modeling methodologies used in these studies vary greatly.

**Table 3:** Summary table for estimates of *R*_0_ in literature.

Authors	Region/country	Time/Year	*R* (95% range)
Chowell et al. (2016)	Colombia	2016/01/17∼2016/04/07	2.2–10.3
Nishiura et al. (2016a)	French Polynesia	W41/2013∼W8/2014	1.8–2.0
	Yap Islands	W17/2007∼W30/2007	4.3–5.8
Nishiura et al. (2016b)	Colombia	W35/2015∼W49/2015	3.0–6.6
Kucharski et al. (2016)	French Polynesia	2013/10/11∼2014/03/28	2.6–4.8
Rojas et al. (2016)	Girardot, Colombia	2015/10/19∼2016/01/22	1.41 (1.15–1.74)
	San Andres Island, Colombia	2015/09/06∼2016/01/30	4.61 (4.11–5.16)
Dinh et al. (2016)	USA/ Florida	2016/05/01∼2016/09/23	0.16 (0.13–0.19)
Majumder et al. (2016a), Majumder et al. (2016b)	Colombia	2015/05/31∼2016/04/16	Smoothed HealthMap: 2.56 (1.42–3.83)
		2015/8/22∼2016/04/16	Traditional (INS) data: 4.82 (2.34–8.32)
Funk et al. (2016)	Yap Islands/ Micronesia	2007/04/15∼2007/07/15	4.8–14
Towers et al. (2016)	Barranquilla, Colombia	2015/10/1∼2015/12/31	3.8 (2.4–5.6)
Gao et al. (2016)	Brazil, Colombia, and El Salvador	2015/05∼2016/02/27	2.055 (0.523–6.300)

A statistical estimation study, using the same French Polynesia Zika case data used in this study, concluded that the maximum likelihood estimate (MLE) of *R*_0_ for French Polynesia range from 1.8 to 2.0 (Nishiura et al., 2016a), which is significantly lower than our result. The Nishiura group also used the same methodology and the confirmed case data in Colombia from week 35, 2015 to yield MLE range of *R*_0_ of 3.0–6.6 (Nishiura et al., 2016b), which is substantially higher than the resulting *R*_0_ from the two waves of cases in Colombia detected in this study, using confirmed case data starting from week 32, 2015 (see Table 3). Although different methodologies were employed, it is surprising that while our use of the Richards model indicates that the outbreak in French Polynesia is much more transmissible than that of Colombia, the studies by Nishiura and others (Nishiura et al., 2016a; Nishiura et al., 2016b) conclude the exact opposite. It is an open question and topic for future study why, while both methodologies are based on the idea of initial exponential growth, the results are so decidedly different.

Another modeling study on transmission dynamics of Zika virus in island populations of six archipelagos during the 2013–2014 outbreak in French Polynesia using compartmental model Kucharski et al. (2016) yields median estimates of the basic reproduction number ranged from 2.6 to 4.8, which is consistent with our fitting results with a range of mean estimates of *R*_0_ of 3.08–5.05 for the six archipelagos using the same datasets (see Table 2), even though different modeling methodologies were used. Note that Kucharski et al. (2016) also calculates the proportion of total number of sentinel sites in French Polynesia to adjust for the variations in number of sentinel sites during the outbreak, which might have affected the case numbers reported.

Our mean estimates of *R*_0_ for land-based populations in Colombia, Guatemala and Suriname range between 1.05 and 1.75, while mean estimates for *R*_0_ of island populations in French Polynesia, Puerto Rico and Saint Martin have a range of 5.70–6.89. Significant gap between transmissibility in island populations as opposed to land-based countries/territories suggests a possibly important role of geographic heterogeneity in the spread of vector-borne diseases. Subsequently, it is interesting to observe that the two island populations in the Caribbean, Puerto Rico and Saint Martin, while differing in transmissibility (*R*_0_) to the three land-based Central American countries, Colombia, Guatemala and Suriname, in fact exhibit similar characteristics (e.g., transmission potential) as fellow island populations in Oceania in their respective Zika outbreaks. Although we note that Puerto Rico and Saint Martin have wide 95% CI ranges for *R*_0_ that overlap the corresponding ranges for Colombia, Guatemala and Suriname.

Previous estimates of *R*_0_ employing vastly different modeling methodologies tend to result in varying estimated values for various affected regions, and in some cases with wide 95% ranges (see Table 3). However, we note that in the only other study that also makes use of the same methodology to estimate *R*_0_ in both land-based and island regions, namely that of Rojas et al. (2016), the resulting estimates are respectively 1.41 (1.15–1.74) for Girardot, a land region in Colombia, and 4.61 (4.11–5.16) for San Andres Island, Colombia. The significantly higher *R*_0_ range for island region when compared to land region is consistent with our own findings of geographical heterogeneity in transmissibility of Zika virus.

It has been proposed that herd immunity for vector-borne diseases such as dengue, Chikungunya and Zika are likely to be about 80% (Cohen, 2016). Subsequently, it has been speculated that Zika transmissions could decrease in the near future, based on observation of historic outbreaks of Chikungunya in Thailand and India in the 1960’s (Cohen, 2016; Franca et al., 2016). Our results showing high initial transmissibility seem to support such assertion.

In Kucharski et al. (2016), it is further estimated that 94% (95% CI [91–97]%) of the total population of the six archipelagos in French Polynesia were infected during the outbreak, concluding that ZIKV may exhibit similar dynamics to dengue virus in island populations, with transmission characterized by large, sporadic outbreaks with a high proportion of asymptomatic or unreported cases. Furthermore, in the study of 2007 ZIKV outbreak in Yap Island (another island population), Duffy et al. (2009) also estimated that 73% of the residents of age three or older have had recent ZIKV infection. With our high estimates of *R*_0_ for 2015–2016 outbreaks in Puerto Rico and Saint Martin, it would be interesting to monitor whether reported Zika infections in this island populations in our study would indeed be substantial reduced, or even disappear in the near future as has been predicted (Cohen, 2016).

Our result has potential implications for planning respective intervention and control policies targeted for island and land-based populations, if one could confidently predict the trending of outbreaks based on its geographic characteristics. Unfortunately, as mentioned earlier, we are unable to satisfactorily fit the Richards model to the respective Zika confirmed case data of other nearby countries/territories in the Americas and Oceania, and hence cannot further ascertain if one could indeed generalize these characteristics we derived from the Richards model fitting of these six countries/territories to the neighboring countries/territories in their respective regions. We also note that the data were reported by each country/territory, with significant differences in surveillance systems and reporting requirements, which must be taken into consideration when making comparisons (Pan American Health Organization, 2016a). These differences could conceivably contribute to differences in our estimation results, in particular that of estimates for *R*_0_.

Limitations of this study, other than that of difference in data quality and availability across the study areas which had been discussed above, pertain mainly to the modeling approach employed. While the Richards model has many advantages in its ease of use and minimum requirement for the data needed for implementation (Hsieh, Fisman & Wu, 2010), its use of cumulative case number both could smooth out stochastic variations in disease incidence data but also introduces auto-correlation in the data, potentially leading to biased high estimates of *R*_0_ as well as errors in parameter estimates and subsequently underestimation of uncertainty in the corresponding confidence intervals (Razum et al., 2003; King et al., 2015; Hsieh, 2015).

In particular, utilizing stochastically simulated data, King et al. (2015) demonstrates that using the cumulative data superficially suggests a higher degree of precision in the estimation of the basic reproduction number *R*_0_, resulting in a potentially overly optimistic estimate of its precision as quantified by the range of its confidence interval. On the other hand, epidemic data by onset or reporting dates are typically subject to ongoing cleaning and correction of onset dates, as well as reporting delays and problems related to missing data and other artificial variations. Using cumulative data has the advantages of smoothing out some of these stochastic variations which might or might not be random in nature. In comparison with using data from French Polynesia and Colombia as examples, using cumulative data in this study does result in slightly better fit then using incidence data (Fig. S1), as shown in Table S2 with adjusted *R*^2^ as goodness of fit measure for comparing model fits using either cumulative or incidence data. However, in this study our interpretation of transmissibility deals mainly with comparison analysis on geographical heterogeneity of *R*_0_ in different countries/territories using the same modeling framework, and hence should remain valid.

##  Supplemental Information

10.7717/peerj.3015/supp-1 Table S1 Table S1 Raw data used for model fit

10.7717/peerj.3015/supp-2 Table S2 Estimates of model parameters using incidence data and the Richards model for daily incidence

10.7717/peerj.3015/supp-3 Figure S1A Model fit using weekly incidence data, French Polynesia

10.7717/peerj.3015/supp-4 Figure S1B Model fit using weekly incidence data, Colombia

## Additional Information and Declarations

### Data Availability

The following information was supplied regarding data availability: The raw data has been supplied as a Supplementary File.
