# Supplementary material for: Temporal patterns and geographic heterogeneity of Zika virus (ZIKV) outbreaks in French Polynesia and Central America
Source: PeerJ. 2017 Mar 21;5:e3015. doi: 10.7717/peerj.3015 (PMC5363263; doi:10.7717/peerj.3015)
Supplement: Table S2 [file peerj-05-3015-s002.docx]

**Table S2. Estimates of model parameters using incidence data and the Richards model for daily incidence which is given by the differential equation:**

**where C’(t) is the time derivative of the cumulative case number C(t).**

**French Polynesia**

| **Time** | **r** | **K** | **A** | **R_0_** | **R^2^** |
| --- | --- | --- | --- | --- | --- |
| W41/2013~  W13/2014 | 0.46  (0.32,0.60) | 9388  (9237,9539) | 0.82  (0.47,1.17) | 2.85  (1.34,4.36) | 0.949 |

**Colombia**

| **Time** | **r** | **K** | **A** | **R_0_** | **R^2^** |
| --- | --- | --- | --- | --- | --- |
| W32/2015~  W43/2015 | 0.23  (0.14,0.32) | 634  (557,711) | 5.43  (-3.28,14.14) | 1.69  (1.19,2.18) | 0.612 |
| W49/2015~  W18/2016 | 0.70  (0.38,1.03) | 5461*  (5350,5573) | 0.50  (0.21,0.79) | 4.97  (0.06,9.88) | 0.954 |

*Estimated total case number of the wave during W49/2015~W18/2016.
