# Supplementary figures and images for: Temporal patterns and geographic heterogeneity of Zika virus (ZIKV) outbreaks in French Polynesia and Central America
Source: PeerJ. 2017 Mar 21;5:e3015. doi: 10.7717/peerj.3015 (PMC5363263; doi:10.7717/peerj.3015)

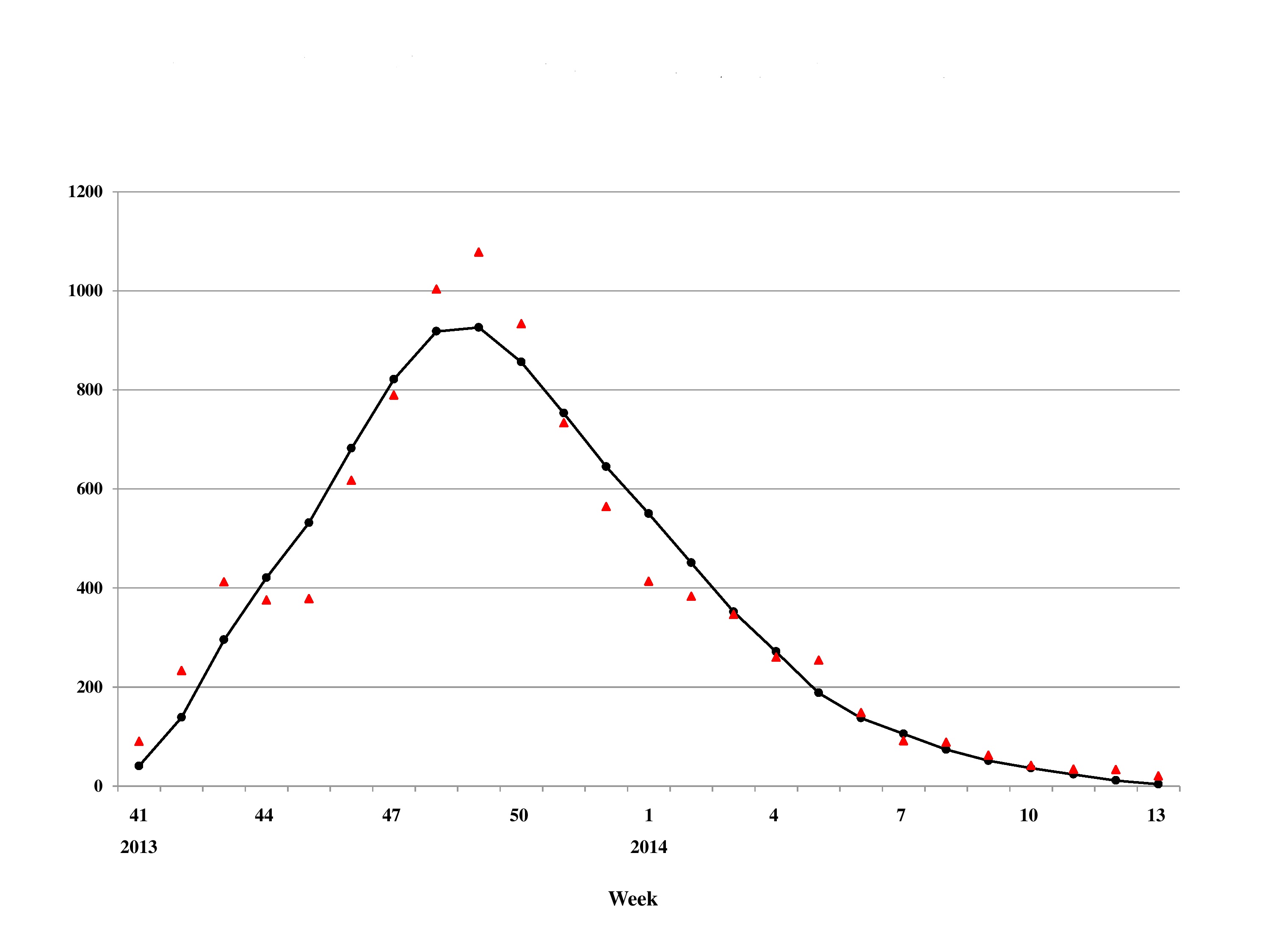

Supplement: Figure S1A [file peerj-05-3015-s003.jpg]

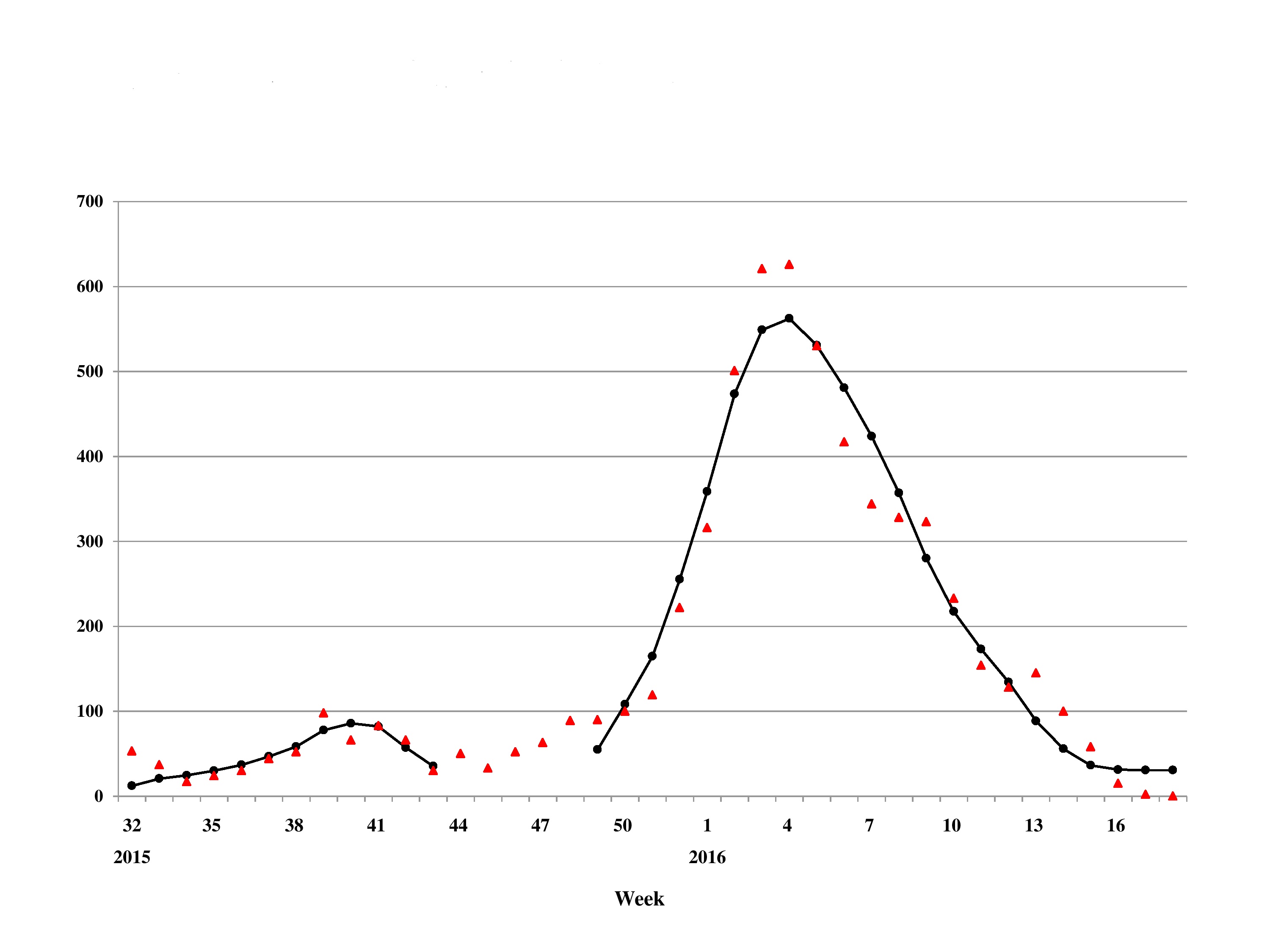

Supplement: Figure S1B [file peerj-05-3015-s004.jpg]
